# Pregnancy or Psychological Outcomes of Psychotherapy Interventions for Infertility: A Meta-Analysis

**DOI:** 10.3389/fpsyg.2021.643395

**Published:** 2021-03-31

**Authors:** Rong Zhou, Yu-Ming Cao, Dan Liu, Jing-Song Xiao

**Affiliations:** ^1^Department of Reproductive Medicine Center, Zhongnan Hospital of Wuhan University, Wuhan University, Wuhan, China; ^2^The Second Clinical College of Wuhan University, Wuhan, China; ^3^Department of Obstetrics and Gynecology, Wuhan Ninth Hospital, Wuhan, China; ^4^Department of Neurology, Zhongnan Hospital of Wuhan University, Wuhan University, Wuhan, China

**Keywords:** infertility, psychological intervention, pregnancy, anxiety, depression

## Abstract

**Background:** The pregnancy and psychological status of infertile couples has always been a concern, but there is no clear evidence for the efficacy of psychotherapy for infertile couples. This study aimed to summarize the current evidence of the effects of psychotherapy on psychological and pregnancy outcomes for infertile couples.

**Method:** We searched Ovid MEDLINE, Ovid EMbase, The Cochrane Library, and Web of Science (ISI) for articles published from 1946 to June 26, 2020. The pregnancy outcomes, psychological outcomes, and acceptability were involved in the study.

**Results:** Overall, 29 studies with a combined total of 3,522 adult participants were included in the meta-analysis. Compared with a placebo, psychotherapy was associated with the pregnancy rate [risk ratio (RR) = 1.43, 95% CI [1.07, 1.93]], total psychological scales associated with infertility [standardized mean difference (SMD) = −0.33 95% CI [−0.63, −0.02]], subsymptoms of psychological scores using the 28-item version of GHQ (including social function [MD = −3.10, 95% CI [−4.30, −1.90]] and depression [MD = −3.90, 95% CI [−5.36, −2.44]], and depression [MD = 3.60, 95% CI [2.25, 4.95]] using the 14-item version of Hospital Anxiety and Depression Scale, but it had no statistically significant association with the other outcomes. In the stratified analyses, the pregnancy rate using assisted reproduction, cognitive behavioral therapy (CBT), and the integrative body–mind–spirit (BMS); total psychological scales associated with infertility using other treatments and more than a month; and anxiety using BMS had significant statistical significance. The funnel plots of all outcomes were approximately symmetrical, and no significant publication bias was found.

**Conclusions:** The study showed that psychotherapy can lead to improvements in the pregnancy rate for infertile patients, especially for patients receiving assisted fertility. In addition, it may help improve total psychological scales associated with infertility and depression. CBT and BMS play an important role in improving rate of pregnancy, and BMS is associated with reducing anxiety. Although psychological interventions had limited effects on the pregnancy outcomes of infertility, our study still recommended that psychotherapies, in particular CBT and BMS, were applied to the therapeutic regimen for infertility, especially for patients receiving assisted fertility.

## Introduction

As the most basic physiological activity of human beings, reproduction plays an important role in stabilizing social units and forming families. Even though fertility and childbirth are a part of life for many couples, ~9–15% of the childbearing population worldwide has infertility (Skakkebaek et al., 2006; Boivin et al., 2007). Infertility, according to The International Committee for Monitoring Assisted Reproductive Technology (ICMART) and the World Health Organization (WHO) Revised Glossary on Assisted Reproductive Technology (ART) Terminology in 2009 (Zegers-Hochschild et al., 2009), was defined as the failure to achieve clinical pregnancy after 1 year or more of unprotected normal sexual behavior. For many couples trying to get pregnant, infertility has become an increasingly serious problem. Although not all couples choose to seek medical assistance, more than 10% of the fertility population had been pregnant with the help of ART (Hart, 2002). Being involuntarily childless and going through various ART procedures impose considerable stress on the couple, and childlessness is often perceived as a life crisis where the emotional strain equals that found for traumatic events (Klonoff-Cohen et al., 2001). At present, this outstanding problem is also gradually by the family, the medical staff, and the social attention.

Current research has found that many factors may lead to infertility, including psychological anxiety and stress. Most couples experiencing infertility will actively use ART to increase their chances of pregnancy and reduce psychological anxiety (Klonoff-Cohen, 2005; Smeenk et al., 2005). Based on a large body of evidence investigating the relevance of psychological interventions and pregnancy, we found that there are related studies confirming that positive psychological interventions increase the chances of pregnancy using ART (Barzilai-Pesach et al., 2006; Ebbesen et al., 2009); the meta-analysis from Frederiksen et al. (2015) and Gaitzsch et al. (2020) suggests that psychosocial interventions for couples in treatment for infertility could be efficacious, both in reducing psychological distress and in improving clinical pregnancy rates. In addition, another meta-analysis from Hämmerli et al. (2009) demonstrated that psychological interventions with the absence of clinical effects on mental health measures were found to improve some patients' chances of becoming pregnant. However, other studies based on meta-analyses took the opposite views (Matthiesen et al., 2011b). Therefore, the magnitude of a possible association between psychological interventions and pregnancy outcomes remains unclear. Improvements in psychological state may also be related to the pregnancy function of infertile patients, which could help indirectly increase the rate of pregnancy. Based on the conflicting above evidences, we performed a systematic review and meta-analysis to investigate the effects of psychological intervention on the pregnancy and psychological outcomes of infertile patients.

## Method

### Search Strategy

We conducted our search using the Preferred Reporting Items for Systematic Reviews and Meta-Analyses (PRISMA) guidelines. We obtained a list of eligible studies that were published in English from 1946 to June 26, 2020, using the following databases: Ovid MEDLINE, Ovid EMbase, The Cochrane Library, and Web of Science (ISI). For the search, Medical Subject Headings (MESH) terms and keywords were used, such as “infertility,” “childlessness,” “IVF,” “ICSI,” “fertility treatment,” “fertility problems,” “assisted reproduction,” “psychological intervention,” “psychosocial intervention,” “social support,” “couples therapy,” “psycho-education,” “internet-based intervention,” and “behavioral therapy.”

### Study Selection

Two reviewers (ZR and JSX) independently assessed abstracts and potentially eligible articles identified during the literature selection. Discrepancies were resolved through discussion. If necessary, a third reviewer (LD) was involved when disagreements could not be resolved.

The following inclusion criteria were used: (1) patients had infertility caused by various reasons; and (2) included a psychological intervention that involved psychotherapy. Types of psychotherapy include cognitive behavioral therapy (CBT); the integrative body–mind–spirit (BMS) intervention and mindfulness provide advice, supportive treatment, and so on; (3) included a placebo control group; and (4) contained at least one of the following outcomes: pregnancy outcomes, psychological outcomes, and acceptability. The outcomes for pregnancy included pregnancy rate and failed pregnancy outcomes, which were designated as ongoing pregnancy, first-trimester miscarriage, biochemical, preclinical, and molar. Psychological outcomes were total psychological scales associated with infertility, anxiety, depression, sexual concern, relationship concern, rejection of a child-free lifestyle, need for parenthood, social concern, depression, psychosom, and social function. The four primary outcomes were pregnancy rate, total psychological scales associated with infertility, anxiety, and acceptability. The other outcomes served as secondary outcomes. In addition, we focused on studies that were randomized controlled trials (RCTs).

The following exclusion criteria were used: (1) patients with polycystic ovary or premature ovarian failure, (2) there were no data available in the original research or the data could not be converted, (3) the study focused on prevention or prevention of relapse, (4) the study was a duplicate publication, and (5) the intervention group included drug treatment.

### Data Extraction and Quality Assessment

Information and data were extracted by two independent authors (ZR and JSX) and proofread, and, if needed, disagreements were arbitrated by a final investigator (LM). With regard to the selection of scales for symptom relief in total psychological scales associated with infertility and anxiety, the priority scale evaluated by clinicians was a self-rated scale. We aimed to compare the difference before and after the change in value, but if those values were not available, we use the final measured value (Higgins and Green, 2011). If a study did not report the data at the end of the study, we chose the most recent data after the end of the study (Gupta, 2011). Sample size was defined as the final sample size for data analysis.

To evaluate the quality of the included studies, two reviewers (RZ and DL) independently assessed the quality of the included studies based on the risk of bias from Cochrane's handbook (Higgins and Green, 2011).

### Statistical Analysis

When analyzing the difference between the binary data, the risk ratio (RR) with 95% confidence interval (CI) was used as the effect size. For continuous data, the mean difference (MD) and standardized MD (SMD) with 95% CI were used (Friedrich et al., 2011). With regard to the heterogeneity of the included studies, the statistical magnitude of *I*^2^ was used (Higgins et al., 2003). Once the result of *I*^2^ was <40, a fixed effect model was adopted. Otherwise, the random-effects model was adopted (Lipsey and Wilson, 2001). To further investigate the heterogeneity of the study, the stratified analyses were employed according to different confounders, including gender, types of psychotherapy, treatment duration, and whether or not to use assisted fertility (Chen et al., 2019). R software (version 3.5.1) was employed for statistical analysis of all outcomes.

## Result

### Search Results

#### Literature Search Results

**Figure 1:**
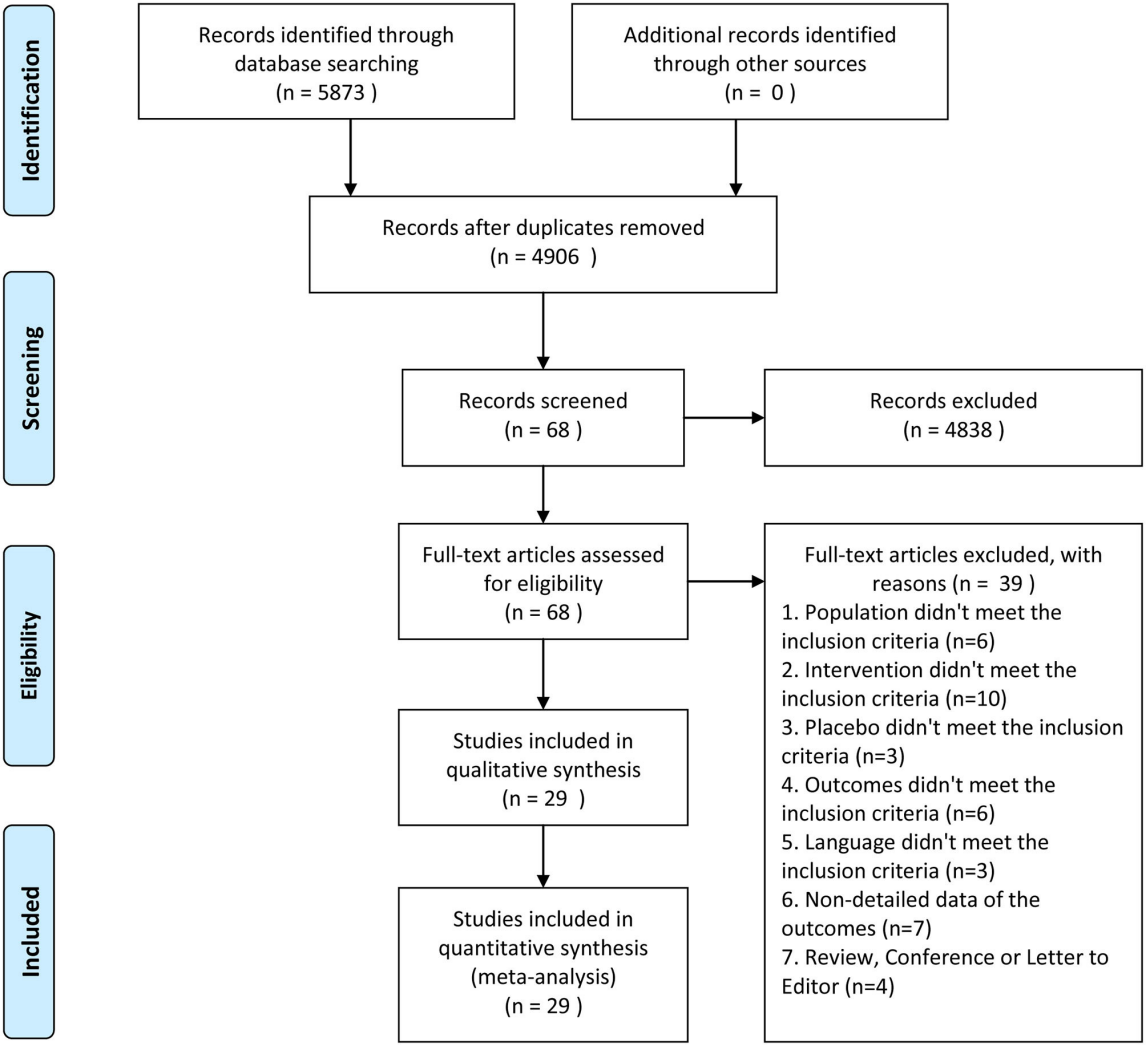
Flow diagram of selection process.

Initially, 5,873 studies were identified from Ovid MEDLINE, Ovid EMbase, The Cochrane Library, and Web of Science (ISI). However, 967 of those were excluded due to duplication, 4,838 studies were excluded by reading titles and abstracts based on the strict inclusion and exclusion criteria, and 68 potential studies were screened for full-text reading. Finally, 29 RCTs with 3,522 individuals were chosen in Figure 1.

#### Study Characteristics and Quality Assessment

The characteristics of 3,522 adult patients from published parallel 29 RCTs (Sarrel and DeCherney, 1985; Domar et al., 2000a,b; Hosaka et al., 2002; Emery et al., 2003; Shu-Hsin, 2003; de Klerk et al., 2005; Chan et al., 2006, 2012; Tuil et al., 2007; Cousineau et al., 2008; Faramarzi et al., 2008; Mori, 2009; Haemmerli et al., 2010; Sexton et al., 2010; Matthiesen et al., 2011a; Skiadas et al., 2011; Gorayeb et al., 2012; Mosalanejad et al., 2012a,b; Domar et al., 2015; Vizheh et al., 2013; Arslan-Özkan et al., 2014; van Dongen et al., 2016; Frederiksen et al., 2017; Nery et al., 2018; Bai et al., 2019; Sahraeian and Lotfi, 2019; Hosseini et al., 2020) were described in the study. The average age ranged from 20 to 40 years. A variety of psychotherapy interventions, including CBT (Domar et al., 2000a,b; Hosaka et al., 2002; Shu-Hsin, 2003; Faramarzi et al., 2008; Haemmerli et al., 2010; Sexton et al., 2010; Gorayeb et al., 2012; Mosalanejad et al., 2012a,b; Domar et al., 2015; Sahraeian and Lotfi, 2019), the BMS (Chan et al., 2006, 2012), and other treatments (Sarrel and DeCherney, 1985; Domar et al., 2000a,b; Emery et al., 2003; de Klerk et al., 2005; Tuil et al., 2007; Cousineau et al., 2008; Mori, 2009; Matthiesen et al., 2011a; Skiadas et al., 2011; Vizheh et al., 2013; Arslan-Özkan et al., 2014; van Dongen et al., 2016; Frederiksen et al., 2017; Nery et al., 2018; Bai et al., 2019; Hosseini et al., 2020), were included in the meta-analysis. Sixteen studies (Shu-Hsin, 2003; de Klerk et al., 2005; Chan et al., 2006, 2012; Tuil et al., 2007; Sexton et al., 2010; Matthiesen et al., 2011a; Skiadas et al., 2011; Gorayeb et al., 2012; Mosalanejad et al., 2012a,b; Domar et al., 2015; van Dongen et al., 2016; Frederiksen et al., 2017; Bai et al., 2019; Hosseini et al., 2020) included patients who received adjuvant therapy. Summary estimates from the meta-analyses are presented in Table 1. The details and overall risks of bias in the study are shown in Supplementary Figure 1.

**Table 1:** Basic characteristics of included studies.

**References**	**Sample**	**Age, mean (SD)**	**No. of female**	**During psychological treatment (weeks)**	**ART**	**Psychological intervention category**	**Psychological score**	**Outcomes**
Arslan-Özkan et al. (2014)	52/53	19−45	NR	2	No	Other	IDS: 39.7 (1.2)/40.5 (10.6); ISE: 22.6 (5.1)/21.9 (5.0); T-FAS: 25.4 (6.8)/19.1 (6.1)	Total psychological scales associated with infertility; acceptability
Bai et al. (2019)	58/59	30.27 (4.14)	156	4	Yes	Other	GAD-7: 6.56 (4.44)/5.91 (3.92)	Anxiety; subsymptoms of psychological scores; acceptability
Chan et al. (2006)	69/115	36.0 (3.28)/35.0 (3.49)	227	4	Yes	BMS	STAI: 47.12/44.62	Pregnancy rate; acceptability
Chan et al. (2012)	110/141	34.32 (3.09)/34.51 (3.42)	339	4	Yes	BMS	STAI: 44.79 (11.47)/43.04 (9.68)	Pregnancy rate; failed pregnancy outcomes; anxiety; acceptability
Cousineau et al. (2008)	49/49	34.53 (4.35)/34.14 (4.29)	99	4	No	Other	STAI: 45.55 (11.64)/45.0 (10.09); FPI: 162.73 (38.04)/160.31 (34.03)	Total psychological scales associated with infertility; subsymptoms of psychological scores; acceptability
de Klerk et al. (2005)	21/19	33.4 (4.7)/33.3 (5.2)	84	3	Yes	Other	DRK: 21 (1.4)/20 (2.0)	Pregnancy rate; total psychological scales associated with infertility; anxiety; acceptability
Domar et al. (2000a)	56/65/63	33.96 (4.32)/33.71 (4.65)/35.19 (4.84)	184	10	No	CBT, Other	NR	Pregnancy rate
Domar et al. (2000b)	56/65/63	33.96 (4.32)/33.71 (4.65)/35.19 (4.84)	184	10	No	CBT, Other	BDI: 8.1 (5.8)/10.2 (6.9)/9.3 (6.1); HRSD: 9.3 (5.4)/8.5 (4.3)/9.5 (5.2)	Acceptability
Domar et al. (2015)	89/77	35.03 (4.18)/34.67 (4.26)	166	NR	Yes	CBT	NR	Anxiety; acceptability
Emery et al. (2003)	86/82	32.1 (3.9)	NA	6	NA	Other	STAI: 36.3 (13.6)/31.3 (9.9)	Anxiety; subsymptoms of psychological scores
Faramarzi et al. (2008)	26/29	28.3 (3.8)/28.4 (5.3)	82	10	No	CBT	Beck scores: 20.1 (7.9)/19.8 (8.5)	Anxiety; subsymptoms of psychological scores; acceptability
Frederiksen et al. (2017)	153/142	31.9 (4.4)/32.9 (4.8)	83/80	14–16	Yes	Other	STAI: 40.3 (10.6)/40.6 (11.8)	Pregnancy rate; anxiety
Gorayeb et al. (2012)	93/95	32.04 (3.94)/32.42 (3.72)	NA	NR	Yes	CBT	NR	Pregnancy rate; acceptability
Haemmerli et al. (2010)	60/64	33.5	103	8	No	CBT	CES-D: 16.7 (11.7)/17.4 (9.7); IDS: 25.2 (3.9)/24.7 (4.9)	Total psychological scales associated with infertility; anxiety; subsymptoms of psychological scores; acceptability
Hosaka et al. (2002)	37/37	34.9/34.7	80	5	No	CBT	HADS: 12.0 (5.6)/11.8 (5.2); POM: 94.1 (30.6)/90.7 (32.7)	Pregnancy rate; anxiety; acceptability
Hosseini et al. (2020)	18/18	25–40	36	1	Yes	Other	NR	Acceptability
Shu-Hsin (2003)	64/68	31.8 (4.2)	132	1	Yes	CBT	SDS: 55.5 (7.2)/57.7 (6.6)	Anxiety; subsymptoms of psychological scores
Matthiesen et al. (2011a,b)	42/40	33.17 (4.15)	NR	6	Yes	Other	COMPI: 14.73 (8.7)/15.00 (9.1)	Total psychological scales associated with infertility; acceptability
Mori (2009)	85/40	30.4 (2.87)/31.3 (2.49)	145	12	No	Other	HADS: 9.7 (5.32)/7.2 (5.77); SF-36: 63.7 (16.97)/68.7 (16.11)	Anxiety; subsymptoms of psychological scores; acceptability
Mosalanejad et al. (2012a)	15/16	20–35	31	17	Yes	CBT	DASS-21: 13.11 (4.76)/6.41 (3.26)	Anxiety; subsymptoms of psychological scores; acceptability
Mosalanejad et al. (2012b)	32/32	20–40	60	13	Yes	CBT	DASS-21: 14 (2.38)/8.87 (3.54)	Anxiety; subsymptoms of psychological scores
Nery et al. (2018)	62/37	37.0 (6.5)/37.4 (5.3)	178	8	No	Other	NR	Acceptability
Sahraeian and Lotfi (2019)	26/26	30.1 (7.16)/29.9 (3.0)	52	6	No	CBT	NR	Acceptability
Sarrel and DeCherney (1985)	20/20	NR	NR	2 h	No	Other	NR	Pregnancy rate
Sexton et al. (2010)	21/22	32.6 (4.8)	43	4	Yes	CBT	SCL-90: 5.0 (1.3)/4.9 (1.2); FPI: 5.3 (1.4)/5.1 (1.4)	Total psychological scales associated with infertility; anxiety; acceptability
Skiadas et al. (2011)	66/65	35.0 (4.2)/34.1 (4.9)	131	1	Yes	Other	PSS: 20.1 (6.5)/18.8 (6.3)	Pregnancy rate; anxiety; acceptability
Tuil et al. (2007)	122/122	36.4/32.7	91	10	Yes	Other	STAI: M: 16.0 (4.4)/15.4 (4.5), F: 16.8 (9.0)/16.3 (5.4); BDI: M: 0.5 (1.2)/0.8 (1.4), F: 1.5 (2.0)/1.3 (2.3)	Anxiety; subsymptoms of psychological scores; acceptability
van Dongen et al. (2016)	61/59	32.0 (4.1)/32.4 (4.8)	120	12	Yes	Other	GEEs: 35 (43.38)/51 (58.67)	Anxiety; acceptability
Vizheh et al. (2013)	43/47	26.9 (4.23)/27.4 (4.65)	100	12	No	Other	NR	Acceptability

*ART, assisted reproductive therapy; IDS, infertility distress scale; ISE, infertility self-efficacy scale; C-STAI, Chinese state-trait anxiety inventory; EBMS, eastern body–mind–spirit; I-BMS, integrative body–mind–spirit; CCRI, cognitive coping and relaxation intervention; CBT, cognitive behavioral therapy; PRCI, positive reappraisal coping intervention; STAI, state-trait anxiety inventory; DRK, daily record keeping chart; COMPI, Copenhagen Multi-center Psychosocial Infertility; SF-36, Short-Form 36-item Health Survey; SCL-90, Symptom Checklist 90-Revised; PHQ-9, Patient Health Questionnaire-9; SDS, Zung's self-administered depression scale; T-FAS, Turkish Version of the Fertility Adjustment Scale; PSS, perceived stress scale; GEEs, generalized estimating equations; FPI, Fertility Problem Inventory; HADS, Hospital Anxiety and Depression Scale; POMS, profile of mood states; NR, not reported*.

### Pregnancy Outcomes

**Figure 2:**
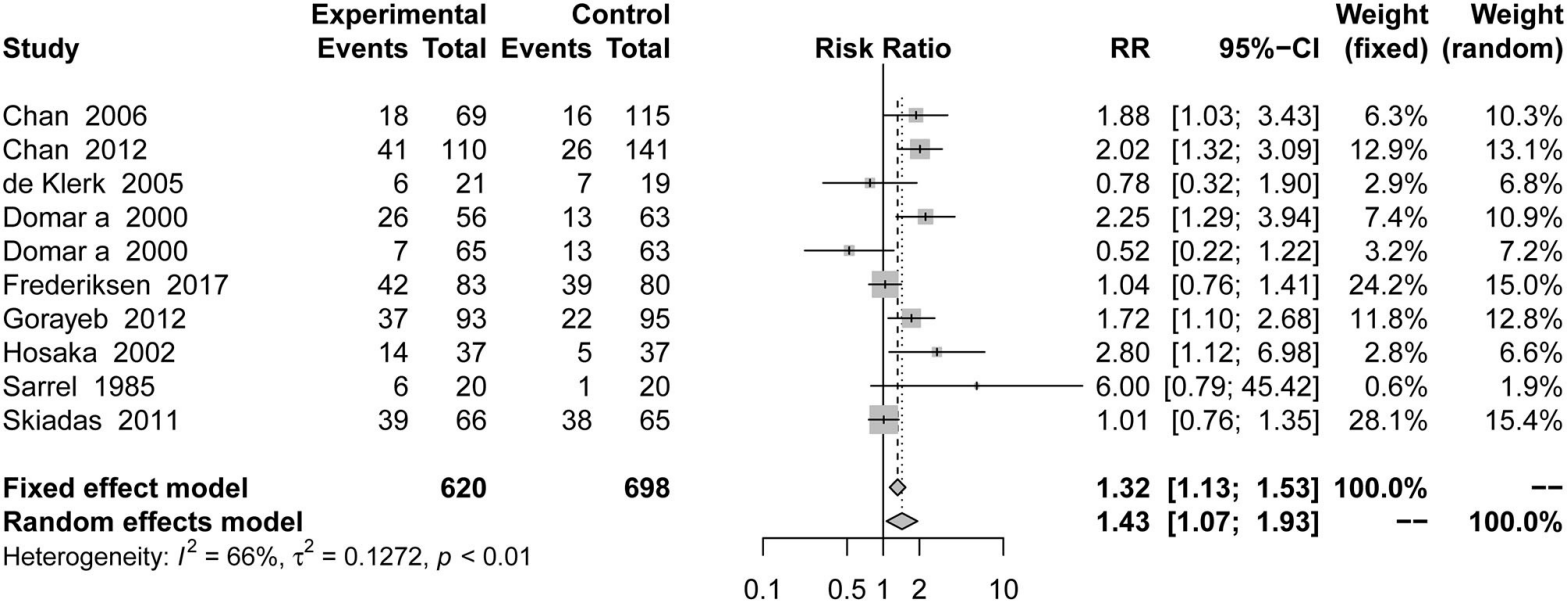
Forest plot of pregnancy rate in pregnancy outcomes.

This outcome was included in 10 studies, with a total of 1,318 patients. Compared with results from the placebo groups, infertile patients who received psychotherapy were more likely to get pregnant than those who received the placebo [RR = 1.43, 95% CI [1.07, 1.93]] based on random-effects model in Figure 2.

The probability of failed pregnancy outcomes, including ongoing pregnancy [RR = 1.31, 95% CI [0.79, 2.19]], first-trimester miscarriage [RR = 0.94, 95% CI [0.29, 2.99]], biochemical (RR = 0.78, 95% CI [0.05, 12.33]], preclinical [RR = 0.78, 95% CI [0.05, 12.33]], and molar [RR = 2.35, 95% CI [0.10, 57.01]], were not statistically significant in Table 2.

**Table 2:** Results of failed pregnancy outcomes.

**Outcomes**	***N***	**RR**	**95% CI**	**P for *I*^2^**
Ongoing pregnancy	251	1.31	[0.79, 2.19]	0.294
First-trimester miscarriage	251	0.94	[0.29, 2.99]	0.911
Biochemical	251	0.78	[0.05, 12.33]	0.860
Preclinical	251	0.78	[0.05, 12.33]	0.860
Molar	251	2.35	[0.10, 57.01]	0.601

*RR, relative risk; CI, confidence interval*.

### Psychological Outcomes

#### Total Psychological Scales Associated With Infertility

**Figure 3:**
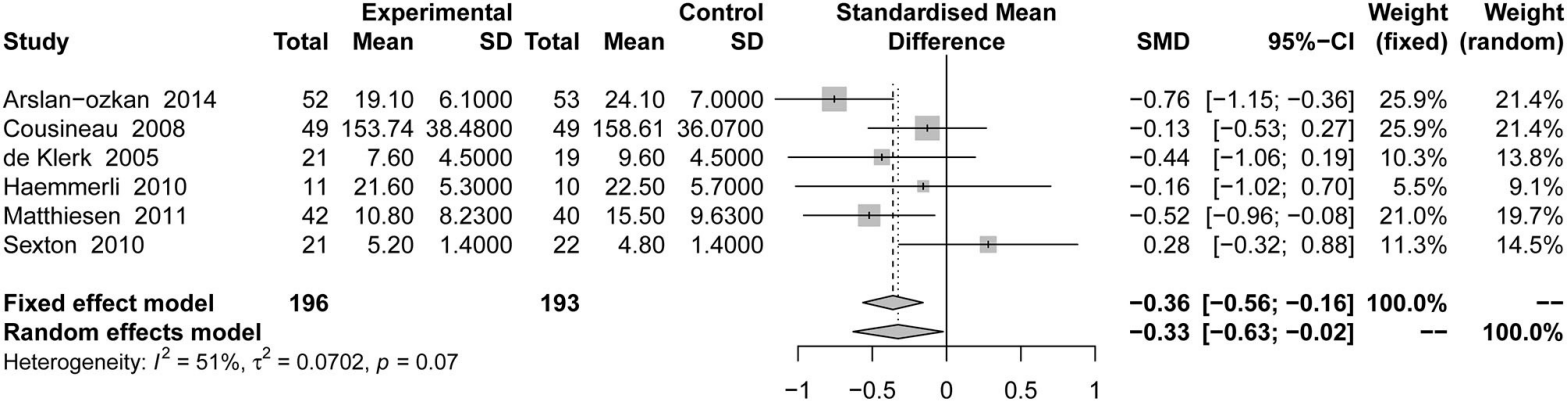
Forest plot of total psychological scales associated with infertility in psychological outcome.

This outcome was included in six studies, with a total of 389 patients. Compared with the placebo groups, psychotherapy was associated with a significant reduction in total psychological scales associated with infertility [SMD = −0.33, 95% CI [−0.63, −0.02]] based on random-effects model in Figure 3.

#### Anxiety

**Figure 4:**
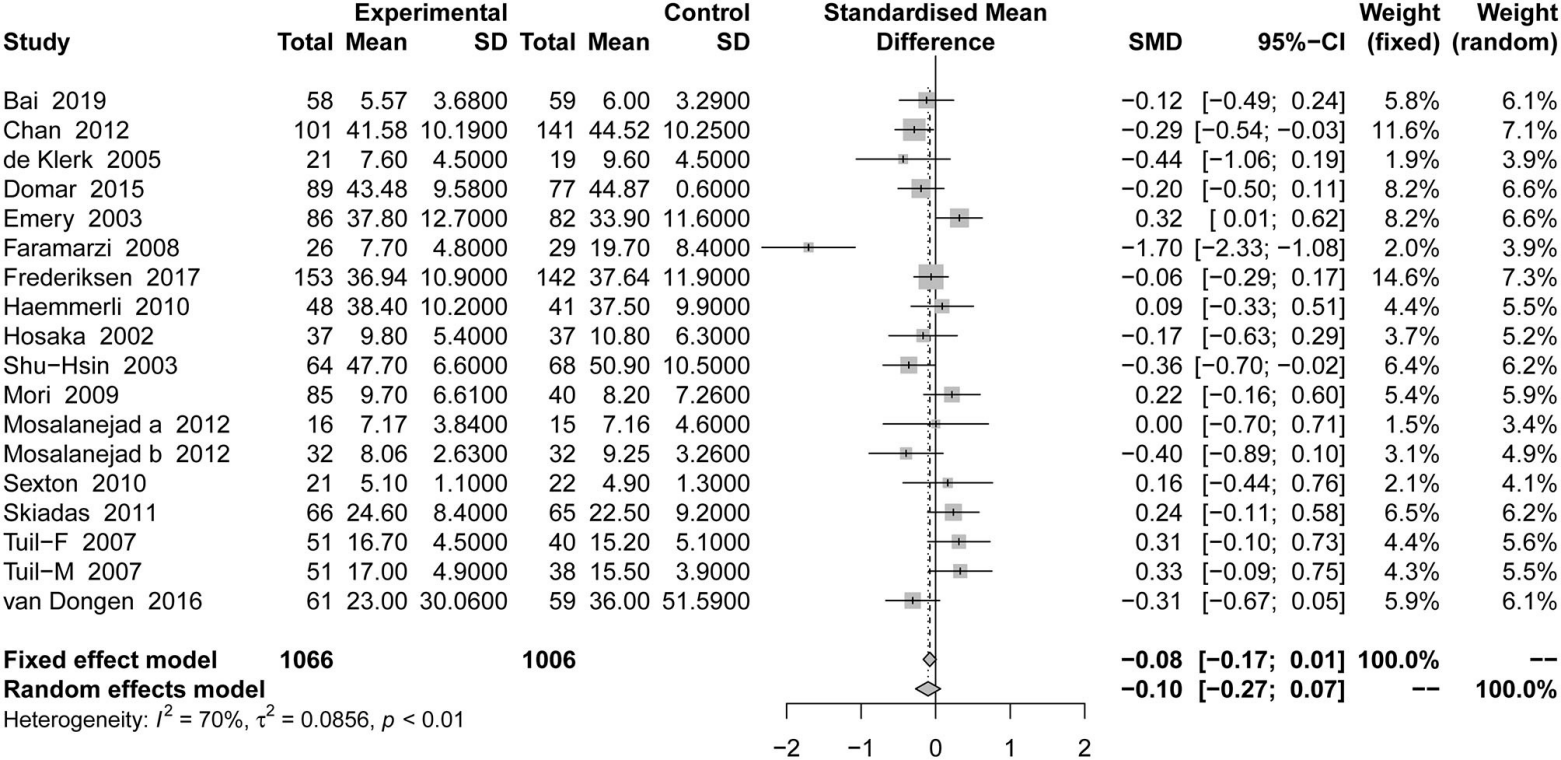
Forest plot of anxiety in psychological outcome.

This outcome was included in 18 studies, with a total of 2,072 patients. Compared with the placebo groups, the effect of psychotherapy on comprehensive anxiety was not statistically significant [SMD = −0.10, 95% CI [−0.27, 0.07]] based on random-effects model in Figure 4.

#### Subsymptoms of Psychological Scores

Subsymptoms of psychological scores using the 46-item version of Fertility Problem Inventory (FPI) [including sexual concern [MD = −0.10, 95% CI [−0.49, 2.92]], relationship concern [MD = 0.85, 95% CI [−4.81, 3.11]], rejection of a child-free lifestyle [MD = −1.56, 95% CI [−5.36, 2.24]], need for parenthood (MD = −0.20, 95% CI [−3.73, 3.33]], and social concern [MD = −0.15, 95% CI [−6.51, 6.81]], psychosom [MD = −2.50, 95% CI [−9.56, 4.55]] using the 28-item version of the General Health Questionnaire (GHQ), and depression using the 14-item version of Beck's Depression Inventory (BDI) [MD = 0.02, 95% CI [−0.19, 0.23]] and Center for Epidemiological Studies Depression Scale (CES-D) [MD = −0.34, 95% CI [−0.70, 0.03]]. The 21-item version of Depression, Anxiety Stress Scale (DASS-21) [MD = 0.11, 95% CI [−0.26, 0.48]], Patient Health Questionnaire-9 (PHQ-9) [MD = −0.31, 95% CI [−0.63, 0.01]], and Zung's self-administered depression scale (SDS) [MD = −0.24, 95% CI [−0.58, 0.11]] were not statistically significant in Table 3. However, other outcomes of subsymptoms of psychological scores using the 28-item version of GHQ [including social function (MD = −3.10, 95% CI [−4.30, −1.90]), and depression (MD = −3.90, 95% CI [−5.36, −2.44])] and depression [MD = 3.60, 95% CI [2.25, 4.95]] using the 14-item version of Hospital Anxiety and Depression Scale (HADS) were statistically significant in Table 3.

**Table 3:** Subsymptoms of psychological scores using different measurements.

**Subsymptoms**	**Rating scale**	**MD**	**95% CI**	**P for *I*^2^**
Sexual concern	FPI-46	−0.10	[−0.49, 2.92]	0.62
Relationship concern	FPI-46	0.85	[−4.81, 3.11]	0.67
Rejection of child-free lifestyle	FPI-46	−1.56	[−5.36, 2.24]	0.42
Need for parenthood	FPI-46	−0.20	[−3.73, 3.33]	0.91
Social concern	FPI-46	0.15	[−6.51, 6.81]	0.97
Depression	BDI	0.02	[−0.19, 0.23]	0.87
Depression	CES-D	−0.34	[−0.70, 0.03]	0.07
Depression	DASS-21	0.11	[−0.26, 0.48]	0.57
Depression	HADS-14	3.60	[2.25, 4.95]	<0.001
Depression	PHQ-9	−0.31	[−0.63, 0.01]	0.054
Depression	SDS	−0.24	[−0.58, 0.11]	0.18
Psychosom	GHQ-28	−2.50	[−9.56, 4.55]	0.49
Social function	GHQ-28	−3.10	[−4.30, −1.90]	<0.001
Depress	GHQ-28	−3.90	[−5.36, −2.44]	<0.001

*FPI, the 46-item version of the Fertility Problem Inventory; HADS, the 14-item version of Hospital Anxiety and Depression Scale; GHQ-28, the 28-item version of the general health questionnaire; BDI, Beck's Depression Inventory; CES-D, Center for Epidemiological Studies Depression Scale; DASS-21, the 21-item version of Depression, Anxiety Stress Scale; PHQ-9, Patient Health Questionnaire-9; SDS, Zung's self-administered depression scale; SMD, standardized mean difference; CI, confidence interval*.

### Acceptability

**Figure 5:**
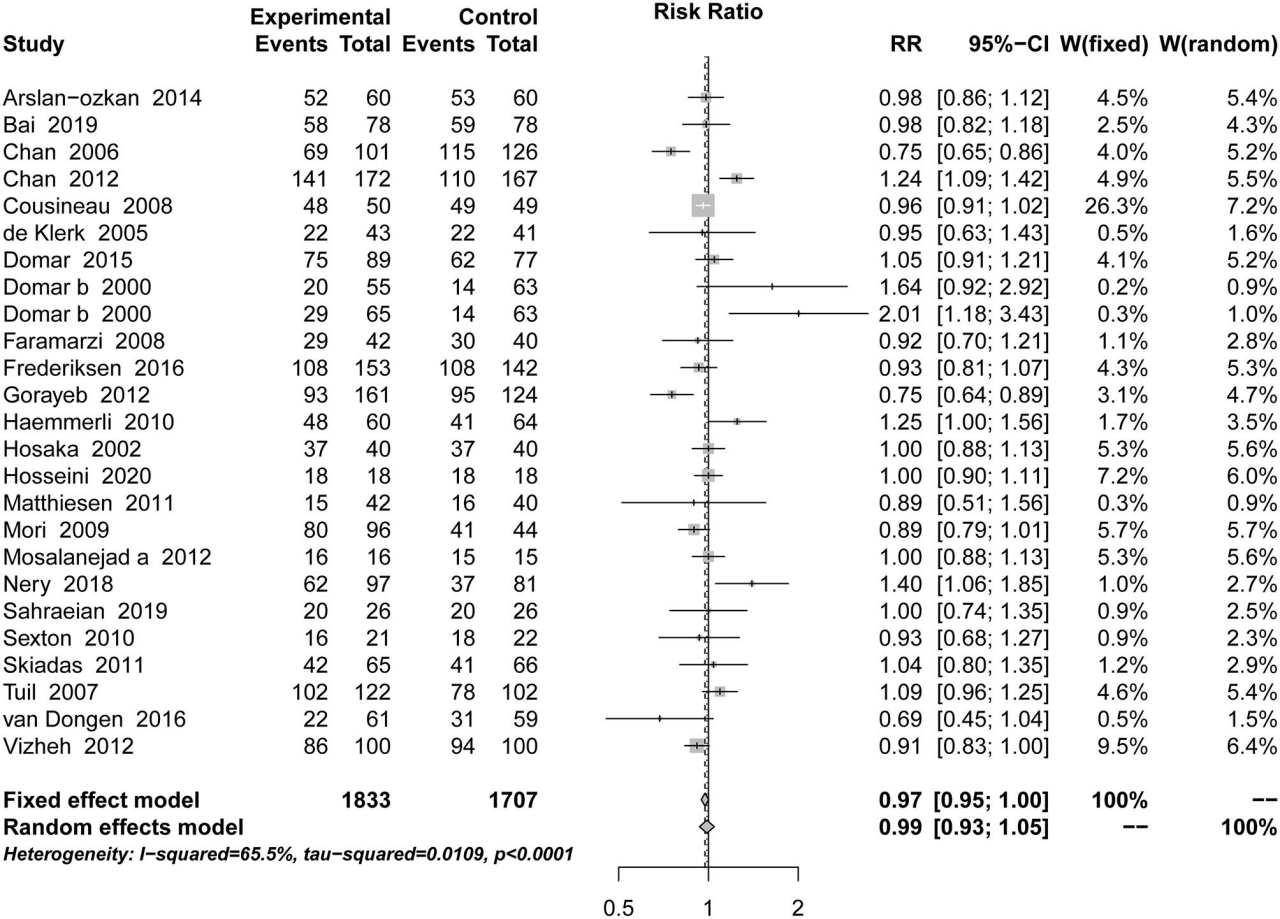
Forest plot of acceptability.

This outcome was included in 25 studies, with a total of 3,540 patients. Compared with the placebo groups, psychotherapy did not lead to more dropouts, and there was no statistically significant difference [RR = 0.99, 95% CI [0.93, 1.05]] based on random-effects model in Figure 5.

### Stratified Analyses

#### Gender

Based on the stratified results including only female, compared with the placebo, Table 4 shows that the psychotherapy intervention was not associated with a significant increase in the pregnancy rate [RR = 1.25, 95% CI [0.95, 1.66]], the total psychological scales associated with infertility [SMD = −0.14, 95% CI [−0.51, 0.23]], anxiety [SMD = −0.14, 95% CI [−0.29, 0.01]], and acceptability [RR = 0.99, 95% CI [0.91, 1.06]].

**Table 4:** Subgroup analysis of primary outcomes based on gender, adjuvant therapy, treatment cycle, and types of psychotherapy.

**Subgroup**	**Results**	**Pregnancy rate**	**Total psychological scales associated with infertility**	**Anxiety**	**Acceptability**
**All**	*N*	1,318	389	2,072	3,540
	SMD/RR, 95% CI	1.43 [1.07, 1.93]	−0.33 [−0.63, −0.02]	−0.10 [−0.27, 0.07]	0.99 [0.93, 1.05]
	P for I^2^	66%	51%	70%	65.5%
**GENDER**					
Female	SMD/RR, 95% CI	1.25 [0.95, 1.66]	−0.14 [−0.51, 0.23]	−0.14 [−0.29, 0.01]	0.99 [0.91, 1.06]
	P for I^2^	56%	45.8%	40.9%	65.6%
**TREATMENT CYCLE**					
Less than a month	SMD/RR, 95% CI	1.18 [0.95, 1.46]	−0.30 [−0.71, 0.11]	−0.16 [−0.37, 0.05]	0.98 [0.89, 1.07]
	P for I^2^	30.3%	70.5%	50.4%	70.9%
More than a month	SMD/RR, 95% CI	1.35 [0.72, 2.51]	−0.44 [−0.84, −0.05]	−0.11 [−0.40, 0.18]	1.02 [0.94, 1.10]
	P for I^2^	76.1%	0%	83.6%	58.7%
**ASSISTED FERTILITY**					
Natural pregnancy	SMD/RR, 95% CI	1.80 [0.74, 4.39]	−0.39 [−0.87, 0.08]	−0.39 [−1.18, 0.41]	0.99 [0.92, 1.07]
	P for I^2^	72.6%	63.8%	92.9%	56.1%
Assisted reproduction	SMD/RR, 95% CI	1.18 [1.002, 1.40]	−0.27 [−0.71, 0.17]	−0.11 [−0.25, 0.03]	0.97 [0.89, 1.06]
	P for I^2^	25.1%	59.4%	45.8%	71.1%
**TYPES OF PSYCHOTHERAPY**					
CBT	SMD/RR, 95% CI	2.00 [1.44, 2.77]	0.14 [−0.36, 0.63]	−0.33 [−0.70, 0.03]	0.96 [0.87, 1.06]
	P for I^2^	0%	0%	81.6%	50%
BMS	SMD/RR, 95% CI	1.49 [1.04, 2.13]	NA	−0.29 [−0.55, −0.02]	0.97 [0.59, 1.59]
	P for I^2^	0%	NA	NA	96.2%
Other	SMD/RR, 95% CI	0.99 [0.81, 1.21]	−0.47 [−0.74, −0.20]	0.05 [−0.12, 0.23]	0.99 [0.94, 1.06]
	P for I^2^	26.8%	41.6%	57.9%	51.4%

*SMD, standardized mean difference; RR, relative risk; CBT, cognitive behavioral therapy; CI, confidence interval; BMS, the integrative body–mind–spirit intervention; NA, not applicable*.

#### Treatment Cycle

Based on different treatment cycles, the placebo group was significantly different from the total psychological scales associated with infertility for more than a month [SMD = −0.44, 95% CI [−0.84, −0.05]]. However, the psychotherapy intervention on pregnancy rate for less than a month [RR = 1.18, 95% CI [0.95, 1.46]] and more than a month [RR = 1.35, 95% CI [0.72, 2.51]], total psychological scales associated with infertility for less than a month [SMD = −0.30, 95% CI [−0.71, 0.11]] and more than a month [SMD = −0.44, 95% CI [−0.84, −0.05]], anxiety for less than a month [SMD = −0.16, 95% CI [−0.37, 0.05]] and more than a month [SMD = −0.11, 95% CI [−0.40, 0.18]], and acceptability for less than a month [RR = 0.98, 95% CI [0.89, 1.07]] and more than a month [RR = 1.02, 95% CI [0.94, 1.10]] were not different in Table 4.

#### Assisted Fertility

In subgroup analyses based on assisted fertility, the placebo group was significantly different from the pregnancy rate using assisted reproduction [RR = 1.18, 95% CI [1.002, 1.40]]. However, the psychotherapy intervention on pregnancy rate using natural pregnancy [RR = 1.80, 95% CI [0.74, 4.39]], total psychological scales associated with infertility using assisted reproduction [SMD = −0.27, 95% CI [−0.71, 0.17]] and natural pregnancy [SMD = −0.39, 95% CI [−0.87, 0.08]], anxiety using assisted reproduction [SMD = −0.11, 95% CI [−0.70, 0.03]] and natural pregnancy [SMD = −0.39, 95% CI [−1.18, 0.41]], and acceptability using assisted reproduction [RR = 0.97, 95% CI [0.89, 1.06]] and natural pregnancy [RR = 0.99, 95% CI [0.92, 1.07]] were not different in Table 4.

#### Types of Psychotherapy

Different psychotherapies were included in the study. In terms of pregnancy rate using CBT [RR = 2.00, 95% CI [1.44, 2.77]] and BMS [RR = 1.49, 95% CI [1.04, 2.13]], total psychological scales associated with infertility using other treatment [SMD = −0.47, 95% CI [−0.74, −0.20]] and anxiety using BMS [SMD = −0.29, 95% CI [−0.55, −0.02]] had significant statistical significance. However, no psychological method was statistically significant in other results in Table 4.

### Publication Bias

Funnel plots of all outcomes were approximately symmetric, and no significant publication bias was found. Funnel plots were performed for acceptability presented in Supplementary Figure 2, but the other outcomes were not shown.

## Discussion

A total of 29 RCTs with 4,010 patients were included in this study to evaluate the efficacy of psychotherapy for infertile patients. The results show that psychotherapy was effective for patients with infertility for both the psychological (e.g., total psychological scales associated with infertility) and pregnancy outcomes (e.g., pregnancy rate) and that there was no difference in tolerance between psychotherapy and placebo.

In contrast to a previous meta-analysis (de Liz and Strauss, 2005) where the pregnancy rate was similar, the current study showed that psychotherapy was associated with a significant increase in the pregnancy rate for infertile patients. Among the much psychotherapy, the advice provided during CBT and BMS had certain therapeutic effects. Other treatments were the only treatments that did not show good results. This may be because supportive therapy does not actually affect the psychological outcomes for patients, and most patients may not think that they need support (de Klerk et al., 2005). Of course, the confounding of other interventions in this study may also lead to negative results, so a specific intervention is worthy of further investigation in the next study. Regarding the intervention cycle, regardless of whether it was more than 1 month or <1 month, it had not significant effect. In terms of gender, both studies with male and female and those with female alone showed an improvement in the pregnancy rate with psychotherapy. However, because there are fewer studies involving the treatment of male infertile patients, the proportion of female is high, and the effectiveness of psychotherapy for male cannot be effectively determined. In terms of assisted reproduction, infertile patients receiving assisted fertility treatment may experience greater psychological trauma, so their pregnancy rate may have a greater increase after receiving psychological treatment. This suggests that patients receiving assisted treatment for infertility may benefit more from psychological treatment. Among the failed pregnancy outcomes, our findings indicated that no correlation between psychological interventions and failed pregnancies.

For psychological outcomes, psychotherapy was effective for treating the pain related to infertility, but no significant effect was found for the symptoms of total psychological scales associated with infertility and other psychological scales. Although these findings are consistent with previous research indicating that infertility can lead to related psychological problems (Peterson et al., 2009), using psychotherapy may not always lead to improvement. Among the much psychotherapy, CBT was not effective for treating the psychological aspects of infertility, except that the BMS had a positive effect on anxiety. Other types of psychotherapy can effectively reduce this overall score in terms of controlling total psychological scales associated with infertility. In the control of anxiety state, only BMS showed a certain efficacy, which may indicate that the mind–body approach is the effective way to solve anxiety, while other psychological interventions may not be the most effective way to solve the comprehensive anxiety problem. In terms of acceptability, none of the psychotherapies performed higher or better than placebo.

The number of part of the intervention type in this study was small. The BMS intervention was supported by only two studies (Chan et al., 2006, 2012), while CBT of psychotherapy was supported by a larger number of samples. The quality of research was generally not high, and there is no high-quality evidence to show that a certain kind of psychotherapy is very effective. The poor quality of the included studies may affect the accuracy of the results, so some of our conclusions still need to be treated with caution. Our stratified analysis also showed that psychotherapy for male is insufficient, but according to the baseline characteristic table, there were few infertility cases associated with male causes. Future studies could focus on male patients to further improve the personalized treatment of infertile patients.

Reviewing the previous meta-studies (Hämmerli et al., 2009), we found that the study of Hämmerli et al. demonstrated that the psychological interventions appear to increase infertile women's chances of becoming pregnant—in particular those who are not receiving ART. It is worth noting that this study found that psychological intervention can improve the pregnancy rate of patients, especially for the patients who received artificial treatment with weak effect. Unfortunately, the research of de Liz and Strauss (de Liz and Strauss, 2005) did not give a clear conclusion on the related issues. Based on the latest study of Frederiksen et al. (2015), their conclusions suggested that psychosocial interventions, in particular CBT and mind–body interventions, are beneficial for reducing distress and for improving pregnancy outcomes of ART. The relevant conclusions are also confirmed by this study. It is worth noting that the definition of mind–body interventions referred to mindfulness, yoga, relaxation, imagery, hypnosis, etc., which is somewhat different from the definition scope of BMS in this study. In other respect, this study also found that psychological intervention did not only increase the pregnancy failure rate but also improved the symptom score of total psychological scales associated with fertility and depression. The findings of the present study provide some evidence in support of integrating psychological interventions as a treatment strategy for infertile patients. Some of the current guidelines did not recommend psychotherapy as a treatment for patients with infertility (Penzias et al., 2020; Wall et al., 2020). This study suggested that psychotherapy can lead to improvements in the pregnancy rate and infertility-related psychological stress of patients with infertility, and it is recommended that psychotherapy be added to the guidelines. Based on the findings of the present study, psychotherapy involving CBT and BMS is recommended as the first choice, especially for the patients who received artificial treatment.

### Advantage and Limitations

We have updated the latest and most comprehensive studies on the comparison of psychotherapy and placebo in the treatment of infertility, and we made corresponding treatment recommendations based on the results of this study. These findings provide a direction for future clinical research and information that can be used for changing guidelines. This study also has some limitations. First, the number of studies was small, and it was difficult to evaluate all current psychotherapies. Second, due to the particularity of psychological intervention, the integrity of the blind method cannot be guaranteed, which will lead to the low quality of the overall study, thus affecting the accuracy and stability of the results. Third, the correlation between studies was not high, so it was difficult to directly compare the advantages and disadvantages of psychotherapy.

## Conclusion

The study showed that psychotherapy can lead to improvements in the pregnancy rate for infertile patients, especially for patients receiving assisted fertility. In addition, it may help improve total psychological scales associated with infertility and depression. CBT and BMS play an important role in improving rate of pregnancy, and BMS is associated with reducing anxiety. Although psychological interventions had limited effects on the pregnancy outcomes of infertility, our study still recommended that psychotherapies, in particular CBT and BMS, be applied to the therapeutic regimen for infertility, especially for patients receiving assisted fertility.

## Data Availability Statement

The original contributions presented in the study are included in the article/Supplementary Material, further inquiries can be directed to the corresponding author/s.

## Author Contributions

J-SX and RZ contributed to the study conception and design, the acquisition of data, and the drafting of the manuscript. RZ, J-SX, and DL contributed to the analysis and interpretation of the quantitative data and the drafting of the manuscript. RZ, J-SX, and Y-MC contributed to the development of critical revision of the final draft. J-SX and RZ contributed to the analysis and interpretation of the descriptive statistics and the revision of the final draft. All authors have read and approved the manuscript.

## Conflict of Interest

The authors declare that the research was conducted in the absence of any commercial or financial relationships that could be construed as a potential conflict of interest.
