# Supplementary material for: Pregnancy or Psychological Outcomes of Psychotherapy Interventions for Infertility: A Meta-Analysis
Source: Front Psychol. 2021 Mar 31;12:643395. doi: 10.3389/fpsyg.2021.643395 (PMC8044306; doi:10.3389/fpsyg.2021.643395)
Supplement: Supplementary file 1 [file Data_Sheet_1.docx]

**Legends of Supplementary Materials**

**Supplementary Figure 1** Risks of bias of included studies

**Supplementary Figure 2** Funnel plots for acceptability

**Supplementary Figure 1** Risks of bias of included studies

**
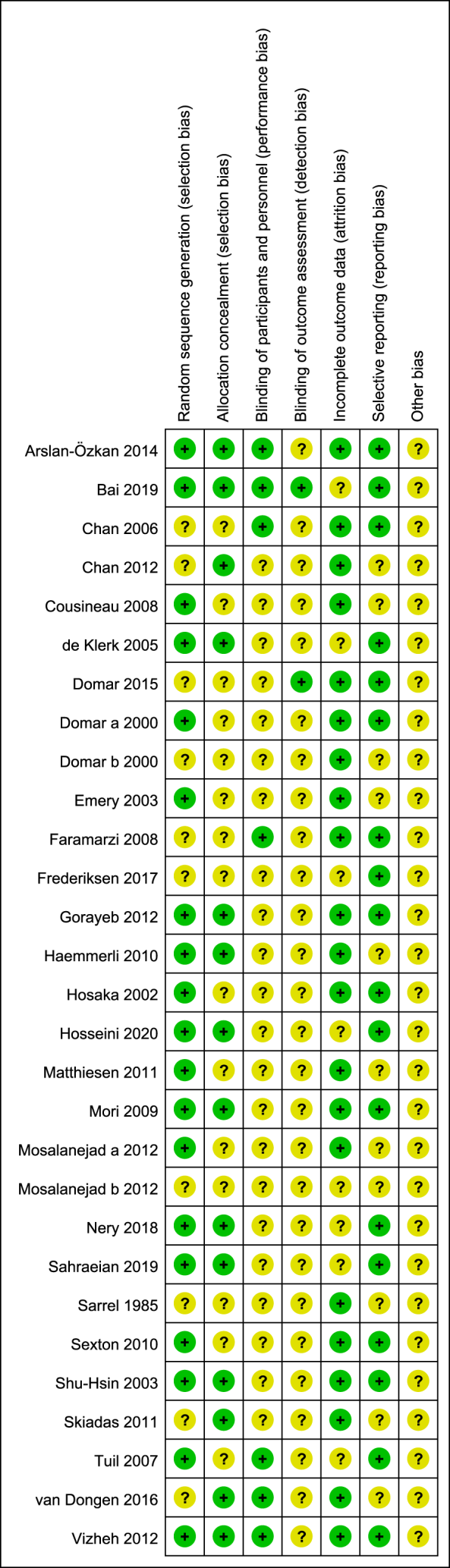
**

**Supplementary Figure 2** Funnel plots for acceptability

**
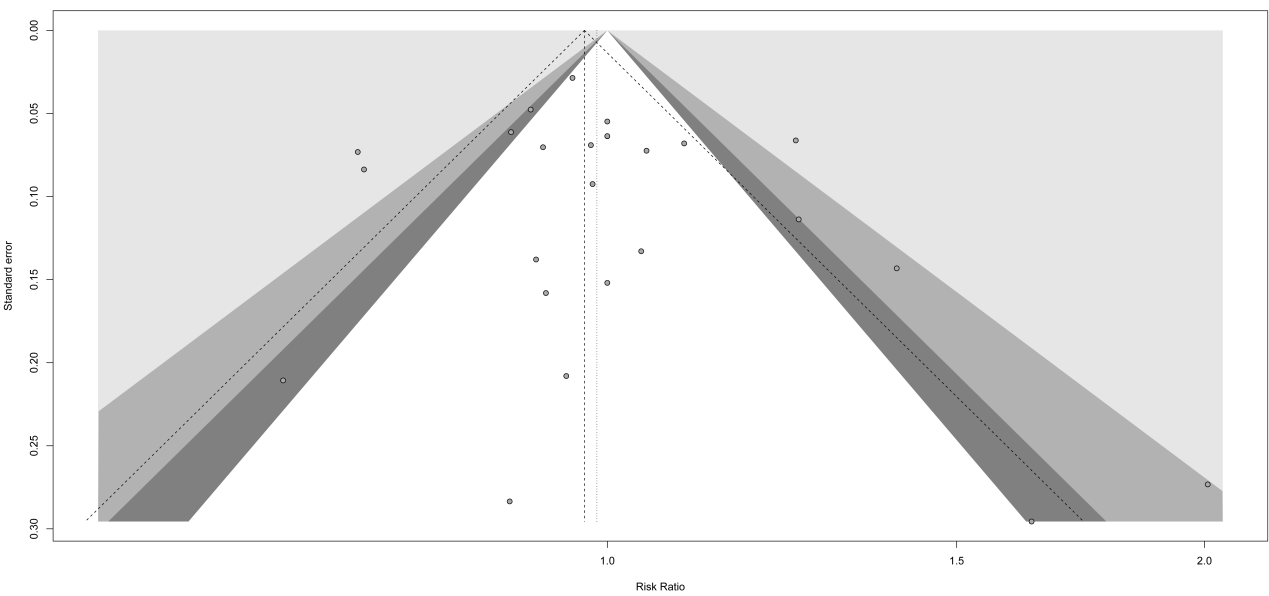
**
